# Supplementary material for: Evaluation of Five Plasma miRNAs as Biomarkers for Minimally Invasive Staging of Liver Fibrosis in β-Thalassaemia Patients
Source: Int J Mol Sci. 2025 Sep 30;26(19):9543. doi: 10.3390/ijms26199543 (PMC12524536; doi:10.3390/ijms26199543)
Supplement: Supplementary file 1 [file ijms-26-09543-s001.zip › Ozkaramehmet_et_al_2025_suppl_data.pdf]

Supplementary data

Evaluation of Five Plasma miRNAs as Biomarkers for Minimally Invasive Staging of Liver Fibrosis in β-Thalassaemia Patients

Sevgi Özkaramehmet[1], Savanna Andreou[1], Kristia Yiangou[2], Soteroula Christou[3], Michalis Hadjigavriel[4], Maria Sitarou[5], Katerina Pyrovolaki[5], Eleni Papanicolaou[6], Christina Flourou[6], Irene Savvidou[3], Panagiotis Boutsikos[3], Alexandra Mendoni[3], Marina Kleanthous[1], Marios Phylactides[1,*], Carsten W. Lederer[1,*]

[1] Molecular Genetics of Thalassaemia Department, The Cyprus Institute of Neurology & Genetics, 6 Iroon
Avenue, Ayios Dometios, 2371 Nicosia, Cyprus; sevgio@cing.ac.cy (S.Ö.); savannaandreou@hotmail.com (S.A.); marinakl@cing.ac.cy (M.K.)

[2] Biostatistics Unit, The Cyprus Institute of Neurology & Genetics, 6 Iroon Avenue, Ayios Dometios,
2371 Nicosia, Cyprus; kristiay@cing.ac.cy

[3] Thalassaemia Clinic Nicosia, Archbishop Makarios III Hospital, 1474 Nicosia, Cyprus; snchrthalcl@cytanet.com.cy (S.C.); savvidesiren@gmail.com (I.S.); p.boutsikos@yahoo.com (P.B.);
alexmendoni1@gmail.com (A.M.)

[4] Thalassaemia Clinic Limassol, Limassol General Hospital, Kato Polemidia, 4131 Limassol, Cyprus;
drmhad@gmail.com (M.H.)

[5] Thalassaemia Clinic Larnaca, Larnaca General Hospital, 6301 Larnaca, Cyprus; msitarou@yahoo.gr (M.S.); k.pyrovolaki@shso.org.cy (K.P.)

[6] Internal Medicine Department, Nicosia General Hospital, Strovolos, 2029 Nicosia, Cyprus; elenipapanicolaou@gmail.com (E.P.); christinafl@hotmail.gr (C.F.)

* Correspondence: mphylact@cing.ac.cy (M.P.); lederer@cing.ac.cy (C.W.L.)

Table S1. Number of valid ΔCt values per fibrosis stage and miRNA after outlier exclusion.

Fibrosis stage	let-7a	miR-21	miR-29a	miR-34a	miR-122
F0-F1	13	13	15	15	15
F2	12	12	12	12	11
F3	6	6	6	5	6
F4	6	7	5	6	5

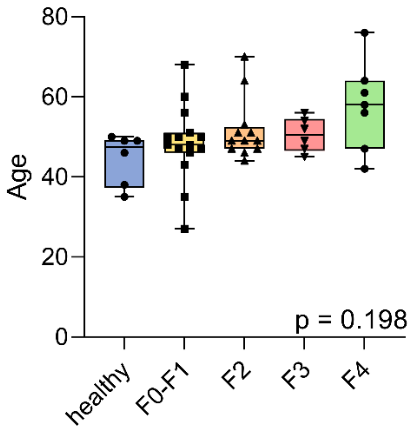

Figure S1. Age distribution across liver fibrosis stages and healthy controls. Each dot represents an individual participant's age. Group sizes were: healthy (n = 6), F0-F1 (n = 15), F2 (n = 12), F3 (n = 7), and F4 (n = 6). Boxplots display the median, interquartile range, and full range within each group. Groupwise comparison was performed using the Kruskal-Wallis test. The corresponding p-value is indicated.

Table S2. Average age and standard deviation for healthy young and healthy old subjects.

Healthy subjects	Number of subjects (n)	Average Age	Standard Deviation
Young	6	28.33	1.97
Old	6	44.50	5.85

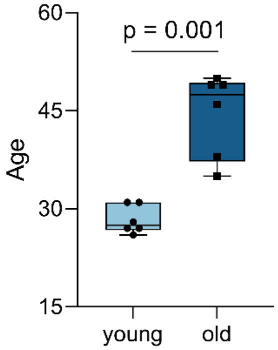

Figure S2. Age distribution of healthy young and healthy old subjects. Boxplots illustrate the age distributions in young (n = 6) and old (n = 6) healthy groups. Boxplots indicate the interquartile range, median, and full data range, with individual data points overlaid. Shapiro-Wilk tests confirmed normality in both groups (p = 0.129 and p = 0.092, respectively). A groupwise comparison by two-tailed independent samples t-test showed a significant difference in age (p = 0.001).

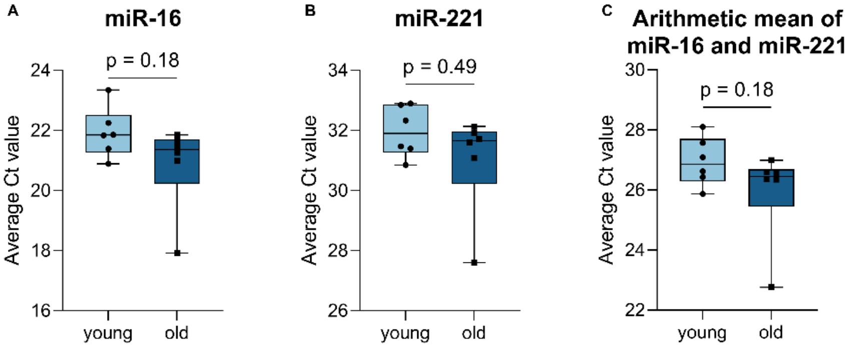

Figure S3. Expression of reference miRNAs miR-16, miR-221, and their arithmetic mean in healthy young and healthy old subjects. Boxplots show the distribution of average Ct values for **(A)** miR-16, **(B)** miR-221, and **(C)** the arithmetic mean of both miRNAs in healthy young (n = 6) and healthy old (n = 6) individuals. Each dot represents an individual sample. Boxplots indicate the interquartile range, median, and full data range. Groupwise comparisons were performed using the Wilcoxon rank-sum test, as assumptions of normality were not met. No statistically significant differences were found between age groups for any of the comparisons (miR-16: p = 0.18, miR-221: p = 0.49, arithmetic mean: p = 0.18).

Table S3. Diagnostic parameters to evaluate the discriminatory power of candidate miRNAs for fibrosis stages (from F0-F1 to F4) in β-thalassaemia patients.

miRNA	Fibrosis stage comparison	AUC*	p-value	OR** (95% CI)	Youden Index***	Optimal Cutoff****	Sensitivity	Specificity
miR-21	F3 vs F4	0.798	0.072	0.064 (0.001-0.815)	0.571	0.140	0.571	1.000
miR-21	F0-F1 vs F3	0.795	0.058	13.461 (1.256-332.421)	0.538	0.180	1.000	0.538
miR-34a	F3 vs F4	0.767	0.168	9.873 (0.774-1063.999)	0.500	7.740	0.500	1.000
let-7a	F2 vs F4	0.750	0.137	0.261 (0.034-1.368)	0.500	4.765	1.000	0.500
let-7a	F2 vs F3	0.743	0.375	0.397 (0.039-2.919)	0.500	4.580	0.833	0.667
miR-29a	F3 vs F4	0.733	0.206	9.197 (0.417-590.672)	0.600	3.095	0.600	1.000
miR-21	F2 vs F3	0.729	0.107	8.912 (0.778-194.570)	0.500	0.705	0.667	0.833
miR-122	F2 vs F4	0.727	0.174	2.212 (0.807-9.147)	0.455	1.920	1.000	0.455
miR-34a	F0-F1 vs F3	0.720	0.149	0.236 (0.021-1.357)	0.533	7.740	1.000	0.533

* AUC, area under the ROC curve, measuring the classifier's ability to distinguish between the two groups. Only analyses with an AUC of 0.7 or higher are shown, sorted by descending AUC score.

** OR, odds ratio; CI, confidence interval.

*** Maximum value of (sensitivity + specificity - 1), reflecting an optimal tradeoff between sensitivity/specificity.

**** Expression value of the miRNA that gives the maximum Youden Index.

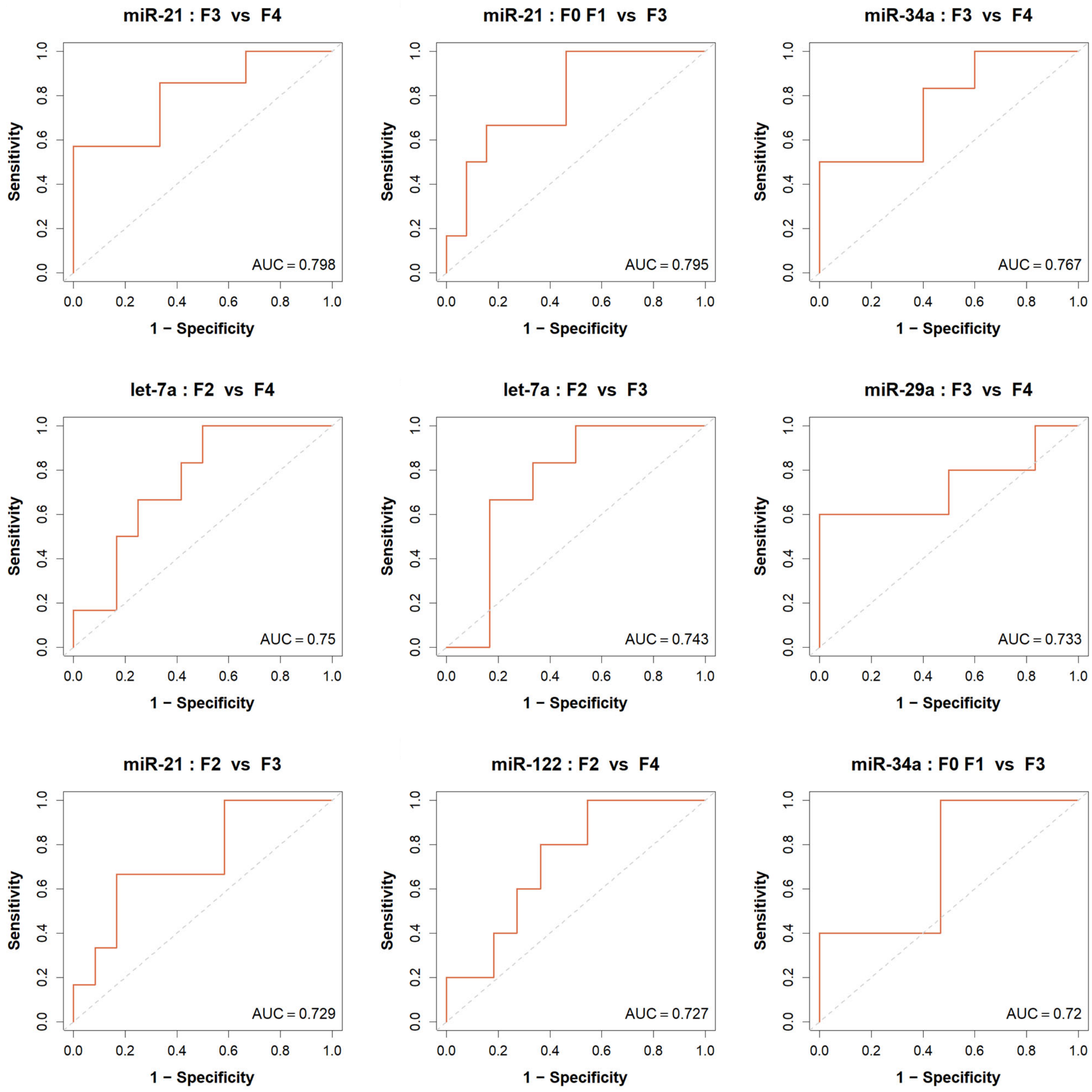

Figure S4. Receiver operating characteristic (ROC) curves for candidate plasma miRNAs distinguishing between liver fibrosis stages in β-thalassaemia patients. ROC curves are sorted by decreasing area under the curve (AUC) values. Reported AUCs range from 0.720 to 0.798 across individual miRNAs (let-7a, miR-21, miR-29a, miR-34a, and miR-122) for the indicated pairs of fibrosis stages. None of the ROC analyses reached statistical significance ($p > 0.05$).

Table S4. Multivariate logistic regression models of combined candidate miRNAs for discrimination between liver fibrosis stages in β-thalassaemia patients.

miRNA combination	Fibrosis stage comparison	AUC*	Youden Index**	Optimal Cutoff***
let-7a + miR-21	F3 vs F4	0.917	0.833	0.697
miR-21 + miR-34a	F3 vs F4	0.900	0.800	0.300
let-7a + miR-34a + miR-122	F0-F1 vs F4	0.885	0.750	0.484
let-7a + miR-34a	F3 vs F4	0.880	0.800	0.578
let-7a + miR-21 + miR-34a	F3 vs F4	0.880	0.800	0.367
let-7a + miR-21 + miR-29a + miR-34a	F2 vs F4	0.875	0.750	0.505
let-7a + miR-21 + miR-34a	F0-F1 vs F3	0.873	0.709	0.512
let-7a + miR-21 + miR-29a + miR-34a	F0-F1 vs F3	0.873	0.709	0.483
let-7a + miR-21 + miR-29a + miR-34a + miR-122	F0-F1 vs F3	0.873	0.709	0.520
miR-21 + miR-29a + miR-34a + miR-122	F2 vs F3	0.873	0.709	0.436
let-7a + miR-21 + miR-29a + miR-34a + miR-122	F2 vs F3	0.873	0.709	0.442
let-7a + miR-21 + miR-29a + miR-122	F2 vs F3	0.864	0.652	0.360
miR-21 + miR-29a + miR-34a	F0-F1 vs F3	0.862	0.646	0.332
miR-21 + miR-34a + miR-122	F0-F1 vs F3	0.862	0.646	0.342
miR-21 + miR-29a + miR-34a + miR-122	F0-F1 vs F3	0.862	0.646	0.380
let-7a + miR-34a + miR-122	F3 vs F4	0.850	0.750	0.617
miR-21 + miR-29a + miR-122	F0-F1 vs F3	0.846	0.615	0.167
let-7a + miR-29a + miR-34a + miR-122	F0-F1 vs F4	0.846	0.667	0.408
let-7a + miR-21 + miR-29a + miR-34a	F0-F1 vs F4	0.841	0.636	0.141
let-7a + miR-21 + miR-34a + miR-122	F0-F1 vs F4	0.841	0.750	0.476
let-7a + miR-122	F2 vs F4	0.841	0.636	0.135
let-7a + miR-21 + miR-122	F2 vs F4	0.841	0.727	0.208
let-7a + miR-34a + miR-122	F2 vs F4	0.841	0.727	0.255
let-7a + miR-21 + miR-34a + miR-122	F2 vs F4	0.841	0.727	0.262
let-7a + miR-21 + miR-34a + miR-122	F0-F1 vs F3	0.836	0.618	0.429
miR-21 + miR-29a	F0-F1 vs F3	0.833	0.590	0.490
miR-21 + miR-122	F0-F1 vs F3	0.833	0.769	0.211
let-7a + miR-21 + miR-29a	F0-F1 vs F3	0.833	0.576	0.501
let-7a + miR-21 + miR-122	F0-F1 vs F3	0.833	0.636	0.232
miR-21 + miR-29a + miR-122	F2 vs F3	0.833	0.727	0.318
let-7a + miR-21 + miR-29a + miR-34a + miR-122	F0-F1 vs F4	0.818	0.667	0.441
let-7a + miR-21 + miR-29a + miR-122	F2 vs F4	0.818	0.636	0.125
let-7a + miR-29a + miR-34a + miR-122	F2 vs F4	0.818	0.636	0.188
let-7a + miR-21 + miR-29a + miR-34a + miR-122	F2 vs F4	0.818	0.636	0.129
let-7a + miR-34a	F2 vs F4	0.817	0.717	0.331
let-7a + miR-21 + miR-34a	F2 vs F4	0.817	0.583	0.149
miR-21 + miR-34a	F0-F1 vs F3	0.815	0.569	0.287
let-7a + miR-29a + miR-34a	F0-F1 vs F4	0.808	0.615	0.141
miR-21 + miR-29a	F2 vs F3	0.806	0.667	0.229
let-7a + miR-21 + miR-29a	F2 vs F3	0.806	0.667	0.258

let-7a + miR-21	F2 vs F4	0.806	0.583	0.360
let-7a + miR-21 + miR-29a + miR-122	F0-F1 vs F3	0.803	0.545	0.160
let-7a + miR-29a + miR-34a	F0-F1 vs F3	0.800	0.600	0.497
miR-21 + miR-122	F3 vs F4	0.800	0.800	0.475
let-7a + miR-29a + miR-34a	F3 vs F4	0.800	0.550	0.493
let-7a + miR-21 + miR-29a	F2 vs F4	0.792	0.583	0.333
let-7a + miR-29a + miR-34a	F2 vs F4	0.792	0.667	0.310
let-7a + miR-29a	F3 vs F4	0.792	0.750	0.523
miR-29a + miR-122	F2 vs F3	0.788	0.636	0.255
let-7a + miR-29a + miR-122	F2 vs F4	0.788	0.636	0.118
let-7a + miR-29a + miR-34a + miR-122	F0-F1 vs F3	0.785	0.600	0.533
let-7a + miR-34a	F0-F1 vs F4	0.785	0.569	0.278
miR-21 + miR-29a + miR-34a	F2 vs F3	0.783	0.583	0.181
let-7a + miR-21 + miR-29a + miR-34a	F2 vs F3	0.783	0.583	0.181
miR-21 + miR-122	F2 vs F4	0.782	0.618	0.342
miR-34a + miR-122	F2 vs F4	0.782	0.636	0.206
let-7a + miR-21	F0-F1 vs F3	0.773	0.576	0.477
let-7a + miR-21 + miR-29a	F0-F1 vs F4	0.773	0.659	0.282
let-7a + miR-29a + miR-122	F2 vs F3	0.773	0.561	0.339
miR-21 + miR-29a + miR-122	F2 vs F4	0.773	0.636	0.227
miR-21 + miR-29a + miR-34a + miR-122	F2 vs F4	0.773	0.636	0.225
miR-21 + miR-34a + miR-122	F2 vs F4	0.764	0.527	0.262
miR-29a + miR-34a	F3 vs F4	0.760	0.400	0.242
let-7a + miR-122	F2 vs F3	0.758	0.409	0.482
let-7a + miR-21 + miR-122	F2 vs F3	0.758	0.485	0.415
let-7a + miR-34a	F0-F1 vs F3	0.754	0.523	0.416
let-7a + miR-34a + miR-122	F0-F1 vs F3	0.754	0.600	0.526
miR-21 + miR-29a	F2 vs F4	0.750	0.467	0.239
miR-21 + miR-34a	F2 vs F4	0.750	0.583	0.343
miR-21 + miR-29a + miR-34a	F2 vs F4	0.750	0.517	0.477
miR-34a + miR-122	F2 vs F3	0.745	0.600	0.473
miR-21 + miR-34a + miR-122	F2 vs F3	0.745	0.600	0.487
let-7a + miR-21 + miR-34a + miR-122	F2 vs F3	0.745	0.600	0.488
miR-21 + miR-122	F2 vs F3	0.742	0.485	0.428
miR-21 + miR-29a	F0-F1 vs F4	0.738	0.462	0.206
let-7a + miR-21	F2 vs F3	0.736	0.500	0.409
let-7a + miR-34a	F2 vs F3	0.733	0.500	0.253
let-7a + miR-29a	F0-F1 vs F4	0.731	0.500	0.484
let-7a + miR-29a	F2 vs F4	0.729	0.583	0.309
let-7a + miR-21 + miR-34a	F0-F1 vs F4	0.727	0.527	0.298
let-7a + miR-34a + miR-122	F2 vs F3	0.727	0.618	0.319
miR-29a + miR-34a + miR-122	F2 vs F3	0.727	0.618	0.400
let-7a + miR-29a + miR-34a + miR-122	F2 vs F3	0.727	0.618	0.391
miR-29a + miR-34a + miR-122	F2 vs F4	0.727	0.545	0.193
let-7a + miR-29a + miR-122	F3 vs F4	0.722	0.667	0.494

miR-34a + miR-122	F0-F1 vs F3	0.720	0.533	0.169
miR-34a + miR-122	F3 vs F4	0.720	0.400	0.256
let-7a + miR-21 + miR-34a	F2 vs F3	0.717	0.433	0.392
let-7a + miR-29a + miR-34a	F2 vs F3	0.717	0.550	0.300
miR-29a + miR-34a + miR-122	F0-F1 vs F2	0.715	0.418	0.376
let-7a + miR-21	F0-F1 vs F4	0.712	0.561	0.326
let-7a + miR-21 + miR-29a + miR-122	F0-F1 vs F2	0.711	0.364	0.535
miR-29a + miR-122	F0-F1 vs F2	0.709	0.418	0.386
miR-21 + miR-29a + miR-34a	F0-F1 vs F4	0.708	0.385	0.181
miR-29a + miR-34a	F0-F1 vs F3	0.707	0.400	0.118
let-7a + miR-21 + miR-122	F0-F1 vs F4	0.705	0.455	0.188
miR-21 + miR-34a	F2 vs F3	0.700	0.433	0.383
miR-29a + miR-34a	F2 vs F4	0.700	0.517	0.424

* AUC, area under the ROC curve, measuring the classifier's ability to distinguish between the two groups. Only analyses with an AUC of 0.7 or higher are shown, sorted by descending AUC score.

** Maximum value of (sensitivity + specificity - 1), reflecting an optimal tradeoff between sensitivity/specificity.

*** Classification threshold from the logistic regression model yielding the maximum Youden Index.

For the complete analysis readout, see Supplementary Excel file. Analyses yielding infinite confidence intervals (0-Inf) were excluded.
